# Towards a simple mathematical model for the legal concept of balancing of interests

**DOI:** 10.1007/s10506-022-09338-3

**Published:** 2022-11-08

**Authors:** Frederike Zufall, Rampei Kimura, Linyu Peng

**Affiliations:** 1 Max Planck Institute for Research on Collective Goods, Bonn, Germany; 2 Waseda Institute for Advanced Study, Waseda University, Tokyo, Japan; 3 Department of Mechanical Engineering, Keio University, Yokohama, Japan; 4 School of Mathematics and Statistics, Beijing Institute of Technology, Beijing, China

**Keywords:** Mathematical model, Weight formula, Balancing of interests, Right to the protection of personal data, Right to access

## Abstract

We propose simple nonlinear mathematical models for the legal concept of balancing of interests. Our aim is to bridge the gap between an abstract formalisation of a balancing decision while assuring consistency and ultimately legal certainty across cases. We focus on the conflict between the rights to privacy and to the protection of personal data in Art. 7 and Art. 8 of the EU Charter of Fundamental Rights (EUCh) against the right of access to information derived from Art. 11 EUCh. These competing rights are denoted by ($i_1$) *right to privacy * and ($i_2$) *access to information*; mathematically, their indices are respectively assigned by $u_1\in [0,1]$ and $u_2\in [0,1]$ subject to the constraint $u_1+u_2=1$. This constraint allows us to use one single index *u* to resolve the conflict through balancing. The outcome will be concluded by comparing the index *u* with a prior given threshold $u_0$. For simplicity, we assume that the balancing depends on only selected legal criteria such as the social status of affected person, and the sphere from which the information originated, which are represented as inputs of the models, called legal parameters. Additionally, we take “time” into consideration as a legal criterion, building on the European Court of Justice’s ruling on the right to be forgotten: by considering time as a legal parameter, we model how the outcome of the balancing changes over the passage of time. To catch the dependence of the outcome *u* by these criteria as legal parameters, data were created by a fully-qualified lawyer. By comparison to other approaches based on machine learning, especially neural networks, this approach requires significantly less data. This might come at the price of higher abstraction and simplification, but also provides for higher transparency and explainability. Two mathematical models for *u*, a time-independent model and a time-dependent model, are proposed, that are fitted by using the data.

**Supplementary Information:**

The online version contains supplementary material available at 10.1007/s10506-022-09338-3.

## Introduction

The extent to which legal thinking and legal concepts could be made operational or usable by technology, has been subject to many approaches in the area of ‘AI and law’ (Aletras et al. 2016; Ashley and Brüninghaus 2006; Bench-Capon and Sartor 2003; Stefanie and Ashley 2003; Katz et al. 2017; Waltl et al. 2017; Zufall et al. 2019). Prior contributions range from conceptional domain modeling (Ashley and Brüninghaus 2006; Bench-Capon and Sartor 2003), to machine learning (Katz et al. 2017; Waltl et al. 2017), to dedicated Natural Language Processing research (Aletras et al. 2016; Zufall et al. 2019). Approaching this task from the perspective of applied mathematics by developing and creating a mathematical model has rarely been explored (Ferrara and Angelo 2012; Kenton 1979; Alexy 2003; Susi 2019). Alexy (2003, 2004) and Susi (2019) proposed rudimentary formulas for balancing rights, but which are limited to defining discrete factors for the decision. This work is well aware of the concerns raised regarding automation of legal decision-making and does not neglect the procedural dimension of law, i.e., its nature as a dialectic process and the need for contestability (Donohue 2018; Hildebrandt 2020; Ronkainen 2011; Selbst et al. 2019). We seek to contribute by investigating the extent to which a mathematical model is able to stand in for a legal assessment performed by a lawyer, while providing methodological transparency and remaining aware of the various contexts of legal decision-making.

We base our investigation on the use case of balancing the rights to privacy and to the protection of personal data in Art. 7 and Art. 8 of the EU Charter of Fundamental Rights (EUCh) (2016) against the right of access to information derived from Art. 11 EUCh (Sect. 2.1). In Sect. 2, we first outline the legal doctrinal framework of balancing competing interests. We explain how the decision of which of these rights outweighs the other one depends on a range of legal criteria, such as the role of the respective person in public life, and the sphere from which the information originates, as well as how much time has been passed since the occurrence of underlying facts (Sect. 2.2). A key step in our methodology is the translation of these legal criteria into mathematical parameters; we refer to these as “legal parameters” which we distinguish from “model parameters”; see Sect. 3. Our mathematical models proposed in Sect. 4 are based on the idea that the outcome *u*—that will determine whether Art. 7 and 8 EUCh or Art. 11 EUCh prevails—depends on values of these legal parameters. To fit our models, data was created by a fully-qualified lawyer and represents typical factual situations where the right to the protection of personal data collides with the right of access to information. We fit these data into a time-independent model and finally further develop this to a time-dependent model suitable to represent the dependence of the outcome on the passage of time (Sects. 4.1 and 4.2). Finally, we evaluate our models and discuss them in comparison to existing approaches in AI and Law and to more complex machine learning algorithms (Sects. 5 and 6).

## The legal concept

### Conflicting interests

A recurring concept in legal systems is the resolution of conflicts between competing interests through balancing (Luizzi 1980; McFadden 1988; David and Sampaio 2018). These interests may be legal, economic or policy-based. They may be those of individuals or of nation-states such as the interest in public security. Prominent examples of individual interests are fundamental rights such as freedom of speech, the right to liberty or privacy rights.

Legally protected interests can exist as fundamental rights at the constitutional or supranational level and then be broken down to more specific legal rules at any level of the hierarchy of norms. In this way, national law may further flesh out conflicts between these rights, ultimately delegating their resolution to courts. For instance, a national constitution may protect the right to free assembly and the right to physical integrity at a more abstract level. National law can then provide details on the conditions under which the right to free assembly may be restricted in favor of physical integrity such as only allowing an assembly under certain security measures.

Regardless of the legal source, conflicts between these rights and competing interests can be legally resolved by balancing them against each other. While legally protected interests vary depending on the legal system, the general concept of balancing is widely recognised (Dreier 2015; Luizzi 1980; McFadden 1988; Schlink 1976). Applied to the process of justifying the interference of one (fundamental) right with another, it is also referred to as the principle of “proportionality” (Barak 2012; David and Sampaio 2018). It may also play a key role in interpreting legal rules as an instrument of teleological reasoning (Sartor 2010).

In order to develop a mathematical model, we build on the following conflict as illustration: Under EU law, an important source for fundamental rights is the EU Charter of Fundamental Rights (EUCh) (2016). It provides in Art. 7 EUCh a *right to privacy* and in Art. 8 EUCh for a *right to the protection of personal data*. These two rights are usually conflated by the European Court of Justice in cases involving the protection of personal data (Lynskey 2014). The Court refers to both rights conjointly and speaks of “the right to privacy, with respect to the processing of personal data” (Court of Justice of the European Union 2014). To simplify, we will mostly refer to the “right to the protection of personal data” and use the term “right to privacy” in the sense that it comprises the right to the protection of personal data as a subset. Furthermore, we do not consider the legal conditions for justifying interferences pursuant to Art. 8(2) EUCh. Art. 7 and Art. 8 EUCh can conflict with the *right to freedom of expression and information* in Art. 11 EUCh. Freedom of expression includes not only the freedom to hold opinions, but also to receive and further disseminate information (*‘access to information’*). The typical example is the disclosure of personal data on the internet as an act of free expression or subject to the right of access to information.

It must be noted at this point that the Charter’s provisions are aimed at the institutions, bodies, offices and agencies of the EU (Art. 51(1) EUCh), and thus, are initially designed to offer individuals protection towards public entities. However, they still affect the horizontal relationship between individuals in that public entities apply them to solve a conflict between individuals by legislation, administrative decision or judicial decision (Hijmans 2016; Reinhardt 2017).

As mentioned above, the abstract stipulation of human rights may be broken down to more specific rules on a lower hierarchal level. The EU General Data Protection Regulation (Regulation 2016) provides in Art. 6(1)(f) a directly applicable provision to justify inferences in the right to the protection of personal data:

Processing shall be lawful only if and to the extent that at least one of the following applies: (f) Processing is necessary for the purposes of the legitimate interests pursued by the controller or by a third party, except where such interests are overridden by the interests or fundamental rights and freedoms of the data subject which require protection of personal data,..

For our purposes, the legitimate interest of third parties would be to have access to information that constitutes personal data. For example, cases could involve obtaining access to information regarding a politician, while this information would at the same time be protected as personal data. Granting access as a form of ‘processing of personal data’ would however only be lawful if the interest in access is not overridden by the data subject’s fundamental right to the protection of personal data. Accordingly, whether or not this condition is met depends on balancing the rights to privacy and to the protection of personal data (Art. 7, Art. 8 EUCh) on one side and access to information (Art. 11 EUCh) on the other side.

### Legal criteria affecting the balancing

The outcome of this balancing varies depending on the circumstances of the case. It is at this point where the abstract conflict of interest becomes concrete: as the law ultimately cannot foresee every possible situation in which these interests might collide, the balancing of interests provides the legal instrument to take into consideration the particularities of each case. In this way, a court ultimately decides which interest(s) outweighs the other(s) in any given case before them.

Looking at past judicial decisions by the European Court of Justice (ECJ) and EU Member States’ jurisprudence can help identify similar approaches in similar cases. And then again, these similarities in ruling or leading cases can be elevated to general guidelines or criteria that will again be considered as settled case law.

For our case—the balancing between the right to the protection of personal data and access to information—these criteria might be: (Guidelines 2014; Court of Justice of the European Union 2014).

- The data subject’s (i.e. the person’s) social status or role in public life.
- The sphere from which the relevant information originated.
- The time that had passed since the occurrence of the underlying facts of that information.
- The risk for the data subject in case of publishing.
- The data subject being a minor.
- The accuracy of the data.
- Etc.

which interest ultimately outweighs the other one depends on the influence of the criteria in the respective case. For instance, access to information relating to a head of a nation-state will, due to the person’s role for the public discourse, be valued higher in comparison to the person’s right to the protection of personal data.

## From legal criteria to mathematical legal parameters

We implement these legal criteria as parameters in our models, called legal parameters. For simplification we only consider ‘status of the person’, ‘sphere of the information’ and ‘time’. These three criteria are the ones that usually stand in the center of the courts’ reasoning on balancing the rights to privacy and to the protection of personal data (Art. 7, Art. 8 EUCh) against access to information (Art. 11 EUCh). This gives us enough case law to inform the data coding (Sect. 3). We assume that potential other criteria are not relevant for our use case or that they are independent parameters with the value 0.5.

Status of the person The data subject’s status relates to what the ECJ has described as “the role played by the data subject in public life” (Court of Justice of the European Union 2014, para. 81, 97, 99). The Court explicitly mentioned this criterion as one that could affect the interest of the public in having access to a respective information. The Article 29 Working Party later illustrated the term by reference to politicians, senior public officials, business-people and members of the (regulated) professions (Guidelines 2014, p. 13). Furthermore, the Working Party stated that the criterion would be broader than the subgroup of ‘public figures’, itself referring to having a degree of media exposure due to their functions or commitments. Here, we understand the criterion as an indicator for the degree of relevance a person is assigned for the public discourse. In cases where the person is already known to the public, his/her status would be considered higher than if the person is completely unknown.

To operationalise this criterion, we define it as the following parameter taking values between 0 and 1:

- $\alpha _p\in [0,1]$: status of the person.

We consider any value approaching 0 as indicating a less relevant role for the public discourse, while the more the parameter approaches 1 the more relevant the person would be considered. The parameter does not contain any information regarding whether the public knowledge is based on the person’s role for political decision or as a person of cultural interest such as artists. We take the following data points as examples to create data:

**Table:** 

	A person that .
$\alpha _p = 0.01$	.. is publicly unknown
$\alpha _p = 0.25$	.. is relatively unknown to the public *(e.g., an ordinary university staff)*
$\alpha _p = 0.50$	.. is to a certain degree known to the public *(e.g., Mayor of Paris)*
$\alpha _p = 0.75$	.. is largely known in public *(e.g., a head of state)*
$\alpha _p = 0.95$	.. is known to nearly anyone on an inter- national level *(e.g., President of the U.S.)*

**Fig. 1:**
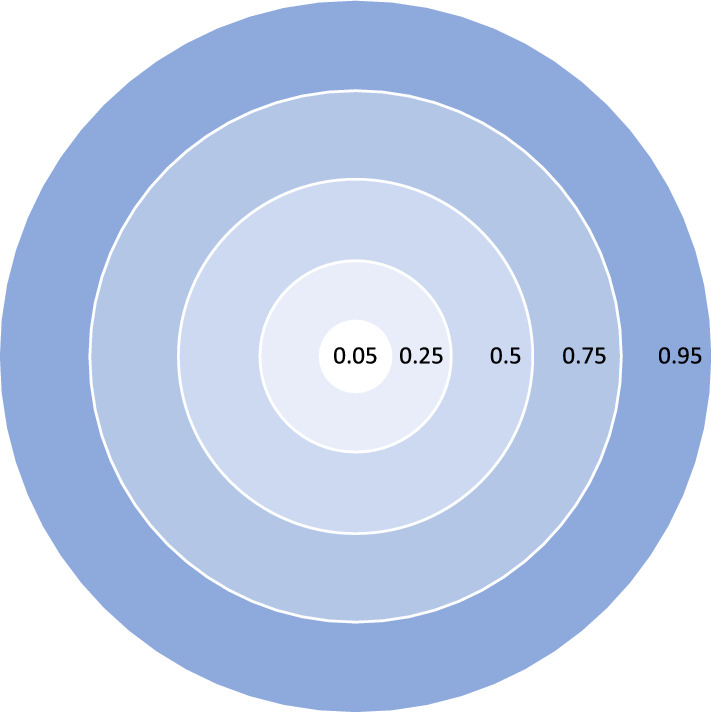
Spheres of information

Sphere of the information Independent of a person’s social status, the information in question can be of a more or less private nature. A common concept to assign a value to this degree is a sphere-model, starting from an inner circle containing the most private information (e.g., health data) followed by information related to family and friends to information related to the social sphere at the outer circle, such as professional life. Fig. 1 illustrates this concept.

We operationalise the sphere of information by the following parameter that is assigned values between 0 and 1:

- $\alpha _s\in [0,1]$: sphere of the information.

The data is created corresponding to $\alpha _s=0.05, 0.25, 0.50, 0.75, 0.95$, respectively.

**Table:** 

$\alpha _s = 0.05$	(e.g., health data)
$\alpha _s = 0.25$	(e.g., family and friends)
$$\alpha _s = 0.50$$	
$\alpha _s = 0.75$	(e.g., professional misconduct)
$\alpha _s = 0.95$	(e.g., committing a major crime)

The idea is to cover information that would be considered as very private to information that would qualify as less private, i.e., originating from a sphere more relevant to the outer world. The sphere, as a legal criterion, has an objective character in the sense that the criterion is independent from the person. Instead, it relates objectively to the nature of the information, regardless of whether it concerns an unknown or a public person.

The higher likelihood of information relating to persons with a public role as justifying access to information relevant to their public roles and activities is being considered at the level of the *outcome*. In turn, if the disclosure of rather private information of a public person is irrelevant for his/her public or professional conduct, the balancing’s outcome would weigh in favor of protecting that information (Art. 7, Art. 8 EUCh).

Time The passage of time in and of itself can be considered as a legal criterion that affects the balancing. The European Court of Justice stated in its Google Spain judgment (2014) that the public interest in a particular piece of information diminishes over the passage of time. This was labeled “the right to be forgotten”. The more time has passed since the occurrence of the facts at issue, the less relevant information about these facts becomes. Dogmatically, then, this perception means the balancing leans increasingly towards the right to data protection over time. Time *t* is nondimensionalised as a legal parameter $\alpha _t$.

- $\alpha _{t}=\frac{t}{T}\in (-\infty ,0]$: a rescaling of time $t\le 0$ with a properly chosen large number $T>0$.

For our models, we set the time of the legal decision at 0, meaning now. The legal decision is made on facts that just occurred ($\alpha _{t}$ = 0), or on facts that occurred in the past, e.g., one year ago ($\alpha _{t} = -\,1$) or 10 years ago ($\alpha _{t} = -\,10$). We consider cases that happened in the following timeframes:

**Table:** 

$$\alpha _{t}= 0$$	(Now)
$$\alpha _{t}= -\,1$$	(1 year ago)
$$\alpha _{t} = -\,3$$	(3 years ago)
$$\alpha _{t} = -\,6$$	(6 years ago)
$$\alpha _{t} = -\,8$$	(8 years ago)
$$\alpha _{t} = -\,10$$	(10 years ago)

Outcome In the above, we have introduced the three criteria “status of the person”, “sphere of information” and “time” independently from each other. For simplicity, in the current study, we assume two outcomes ($i_1$) right to privacy and ($i_2$) access to information, whose indices are respectively denoted as $u_1$ and $u_2$ such that

$$\begin{aligned} u_k\in [0,1],\quad k=1,2, \text { subject to } u_1+u_2=1. \end{aligned}$$

Therefore, it is sufficient to use a single parameter $u\in [0,1]$ as the balancing outcome to model the dependencies of the balancing. For instance, one may assume that the more *u* approaches 0, the more weight is given to ($i_1$) data protection, while the more *u* approaches 1, the more weight is given to ($i_2$) access to information. In the first case, a court would be more likely to rule that the disclosure of personal data is unlawful (balancing in favor of Art. 8 EUCh); in the second that the disclosure is legal (balancing in favor of Art. 11 EUCh).

Data coding Based on the above criteria, a dataset is created that serves as training data for the models proposed in Sect. 4.

The data are hand-coded by a fully-qualified German lawyer, with the necessary qualification for a judge. This is not saying that the data coding is infallible and without error: another lawyer with the same qualification may come to different conclusions on specific cases. In legal practice, too, opinions of lawyers and of judges may differ to a certain degree, but still share a common basis in settled case law and standards.

Accordingly, the data points are based on standards inferred from the relevant case law. As an underlying use case, we take the publication of personal data as information on the internet. It was ensured that the values provide internal consistency. More specifically, the values reflect the standards from case law of how the sphere of information (ranging from health data to more ‘public’ data) as well as the status of the person affect the balancing. For instance, the fact that health data ($\alpha _s$ = 0.05) enjoys utmost protection under Art. 9 GDPR is reflected by not allowing a value over 0.49 for the outcome *u*, even for a person with a highly relevant status for the public discourse ($\alpha _p$ = 0.95). In other words, even a head of state would be protected against the publication of his/her health data at any time (outcome *u* = 0.4 for current data, i.e. “now”). Another example is that the data reflects the legal assumption that the passage of time affects the balancing in favor of access to information, but in a nonlinear way: while it legally makes a huge difference whether the facts at issue had just occurred or occurred 3 years ago (outcome *u* = 0.5 and 0.35 for $\alpha _s = 0.5$; $\alpha _p = 0.5$), after a certain period of time the impact of time becomes smaller. For instance, whether 8 or 10 years have passed since the occurrence is both having a similar impact in favor of access to information (outcome *u* = 0.21 and 0.2 for $\alpha _s = 0.5$; $\alpha _p = 0.5$).

Furthermore, this is a simplification for our models. Existing and even hypothetical cases only allow for generalisation to a certain degree. Real cases naturally depend on more than just three criteria and might even differ from past cases and require the creation of new criteria.

That being said, for the purpose of creating data to fit our models, we fixed the above described values for our legal parameters at 0.01, 0.25, 0.50, 0.75, 0.95 for $\alpha _p$ (status of the person); 0.05, 0.25, 0.50, 0.75, 0.95 for $\alpha _s$ (sphere of information) and the six points in time ranging from now to 10 years ago for $\alpha _t$.

The data are created for all combinations of these data points, i.e., with 150 sets of outcomes *u*. We omitted the values 1 and 0 because legally it is difficult to determine “absolute” privacy and “absolute” access to information. Table 1 illustrates examples from the dataset.

**Table 1:** Examples of data for *u* (outcome) for given values of $\alpha _p$ (person), $\alpha _s$ (sphere) and $\alpha _t$ (time)

$\alpha _p$	$\alpha _s$	$\alpha _t=0$	$\alpha _t=-\,1$	$\alpha _t=-\,3$	$\alpha _t=-\,6$	$\alpha _t=-\,8$	$\alpha _t=-\,10$
0.95	0.05	0.40	0.30	0.15	0.01	0.001	0.00
0.25	0.95	0.65	0.60	0.50	0.45	0.40	0.35

## The mathematical models

For any given piece of information, the purpose of the models is to determine whether ($i_1$) right to privacy outweighs ($i_2$) access to information or vice versa. To summarize, the parameters are defined as follows:

**Table:** 

$\alpha _p\in [0,1]$	Status of the person
$\alpha _s\in [0,1]$	Sphere of the information
$\alpha _{t}=\frac{t}{T}\in (-\infty ,0]$	A rescaling of time $t\le 0$ with a properly chosen large number $T>0$
$u_k\in [0,1]$ subject to $u_1+u_2=1$	Index for ($i_k$), $k=1,2$

The final decision can be made by comparing the values of $u_1$ and $u_2$. However, the constraint $u_1+u_2=1$ allows us to define one single index to fulfill the task. This is the outcome or the output *u*, which is a function of the legal parameters $\alpha _p$, $\alpha _s$ and $\alpha _t$. The final decision, namely whether ($i_1$) right to privacy or ($i_2$) access to the information dominates, is made via the comparison with a prior given threshold value $u_0\in [0,1]$. Without loss of generality, we assume that when $u\le u_0$, ($i_1$) dominates, and otherwise, ($i_2$) dominates.

Accordingly, for outcome values (*u*) lower than the threshold value ($u_0$), the balancing leans towards the right to the protection of personal data (Art. 8 EUCh), while outcome values higher than or equal to the threshold represent a prevailing right of access to information (Art. 11 EUCh). Though a threshold value of $u_0$ = 0.50 might appear intuitive, a varying threshold has the advantage that a preponderant preference of one interest over the other one can be modeled, for instance if legal systems tend to assign per se more weight to one of the interests.

### A time-independent mathematical model

For simplicity, we first propose a simple quadratic model for each (rescaled) year $\alpha _t$ respectively as follows

4.1 $$\begin{aligned} u(\alpha _p, \alpha _s) = c_{00}+ c_{10}\alpha _p + c_{01} \alpha _s + c_{20} \alpha _p^2 +c_{11}\alpha _p\alpha _s+ c_{02} \alpha _s^2\, , \end{aligned}$$

where $c_{00}, c_{01}, \ldots$ are to be determined using the given dataset for each year separately. Since the legal parameters are defined within the domain [0, 1], we simply assumed higher order contributions are negligibly small, but one can consider such terms in order to increase the accuracy of the model if a sufficiently large number of data points is given. Note that in the mathematical model, the legal parameters $\alpha _p$, $\alpha _s$ and $\alpha _t$ are model arguments while $c_{00}, c_{10}, \ldots$ serve as model parameters.

Having determined the coefficients by the dataset, the model parameters of the linear terms, $c_{00}$, $c_{10}$, and $c_{01}$, represent the importance of the legal parameters in a linear plane. In legal terms, they stand in for the importance the respective legal parameter has for the outcome of the balancing decision. The symmetric coefficient matrix *K*, defined as $u(\alpha _p, \alpha _s) = c_{00}+ c_{10}\alpha _p + c_{01} \alpha _s +\frac{1}{2}v^T K v$ with the vector $v = (\alpha _p, \alpha _s)^T$, reflects local geometric properties of the outcome function *u*, e.g., convexness or concaveness. Consequently, it would also reflect the structure of the legal concept of balancing of interests. In addition, the symmetric matrix *K* can always be diagonalized by a matrix *P*, and the diagonalized matrix $D=P^TK P$ represents the sensitivity of the quadratic terms in the direction of the new vector $P^T v$, which is a linear combination of the legal parameters.

In cases where the person is completely unknown ($\alpha _p$ = 0) and the sphere of information would be absolutely private ($\alpha _s$ = 0), we consider the outcome of the balancing being ultimately in favor of data protection (*u* = 0) for any point in time. We thus impose the reasonable assumption

4.2 $$\begin{aligned} u(0,0) = 0 \text { for all } \alpha _t \,. \end{aligned}$$

This fixes one of the coefficients, i.e., $c_{00} = 0$ for all $\alpha _t$.

Furthermore, for cases where a person is known to absolutely anyone in the world ($\alpha _p$ = 1) and the sphere of information would be absolutely public ($\alpha _s$ = 1), we consider the outcome of the balancing being ultimately in favor of access to information (*u* = 1) for any point in time. We thus impose another assumption for the maximum value of $\alpha _p$ and $\alpha _s$:

4.3 $$\begin{aligned} u(1,1) = 1 \text { for all } \alpha _t\,, \end{aligned}$$

leading to that

4.4 $$\begin{aligned} c_{10} + c_{01} + c_{20} + c_{11} + c_{02} = 1 \end{aligned}$$

holds for all $\alpha _t$.

The proposed model can be regarded as a linear optimisation problem for which the coded data can be used to determine the above coefficients, i.e. model parameters. Thus, we fit this function with the coded data (Sect. 3. by using Mathematica (Wolfram Research Inc. 2020); the algorithm is based on the theory of linear least squares. In Table 2, the optimal coefficients (denoted by $c^*$), e.g., model parameters, are listed for each year.

**Table 2:** Fitted model parameters for each year using the model (4.1)

$\alpha _t$ (year)	$c_{01}^*$	$c_{10}^*$	$c_{02}^*$	$c_{20}^*$
0	0.756269	0.218749	− 0.144324	0.181876
$-$ 1	0.655165	0.0286861	− 0.088864	0.301803
$-$ 3	0.429315	− 0.159663	0.00774652	0.390121
$-$ 6	0.184965	− 0.174577	0.15114	0.253607
$-$ 8	0.129208	− 0.241208	0.163708	0.30786
$-$ 10	0.0662971	− 0.295998	0.185813	0.364145

**Fig. 2:**
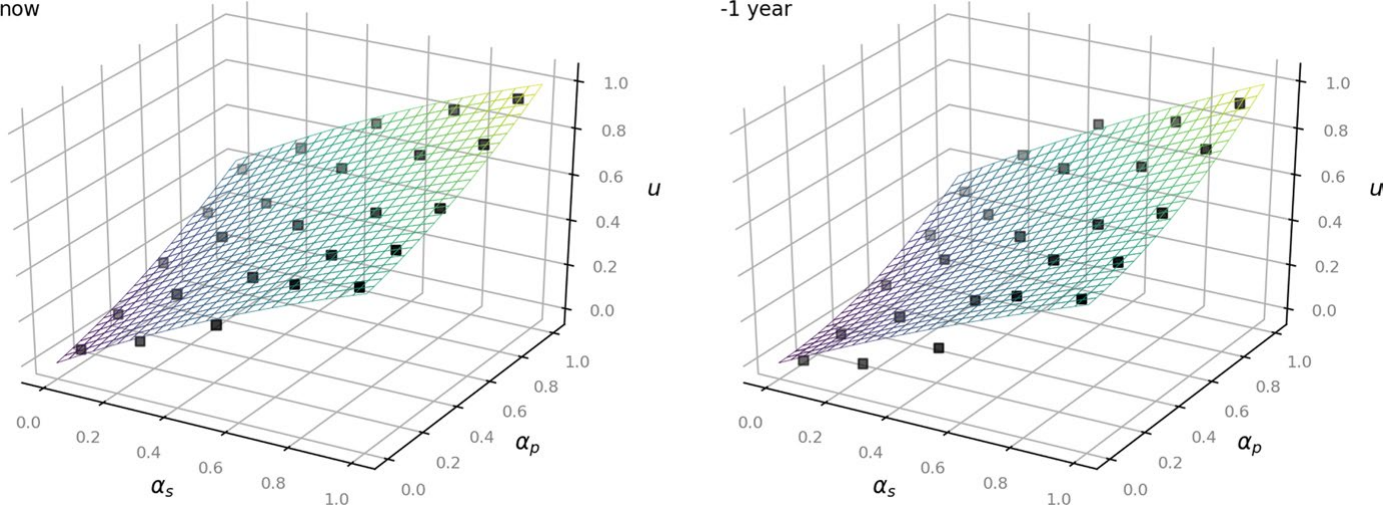
Left: now. Right:—1 year for the time-independent model (4.1)

**Fig. 3:**
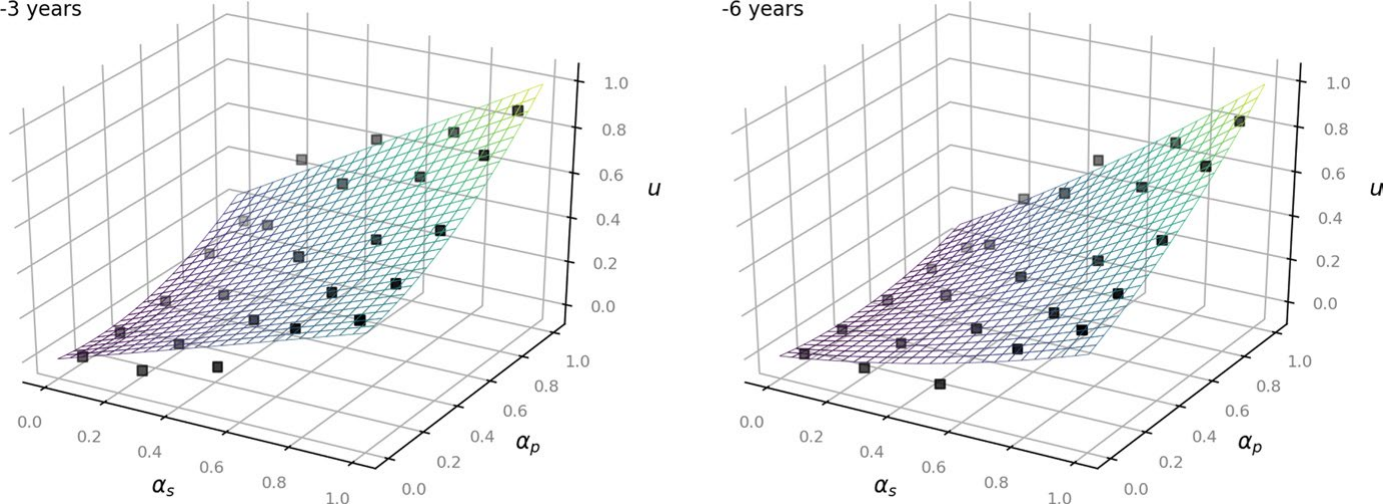
Left:—3 years. Right: —6 years for the time-independent model (4.1)

**Fig. 4:**
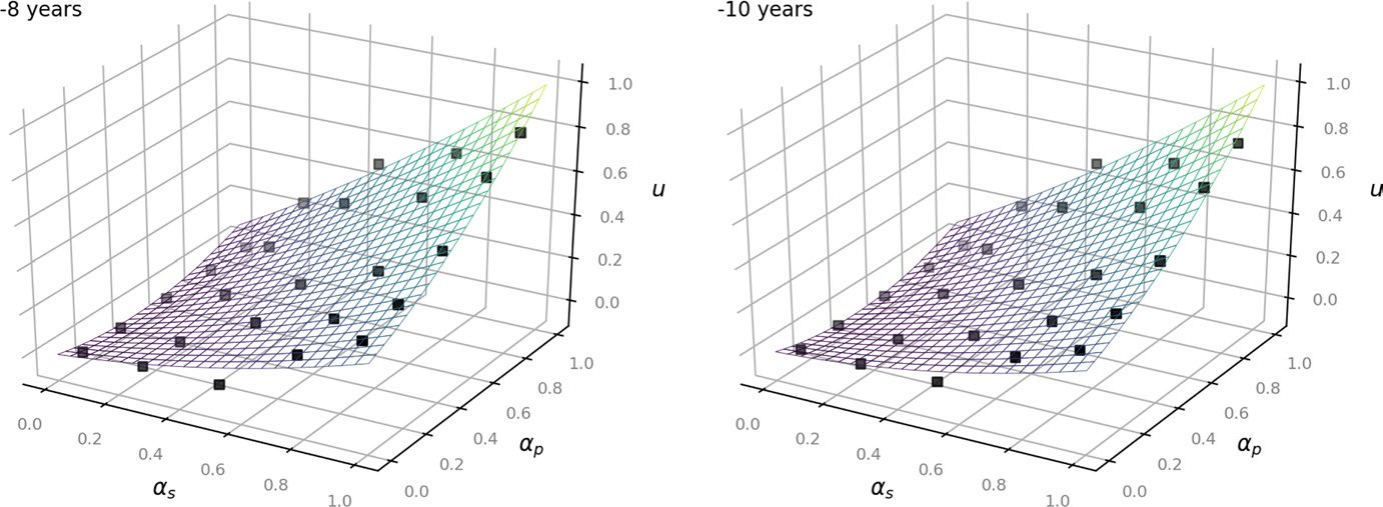
Left:—8 years. Right:—10 years for the time-independent model (4.1)

The following Figs. 2, 3 and 4 show the given data points from the dataset as black dots and the fitted model as a plane. They illustrate how the outcome *u* increases depending on the values of $\alpha _p$ and $\alpha _s$: the higher the status of the person, the less private the information and the less time has passed since the occurrence of the underlying facts, the more the balancing leans towards access to information (Art. 11 EUCh). In turn, more private information of a rather unknown person that occurred several years ago affects the outcome to lean towards data protection (Art. 7, Art. 8 EUCh).

One weakness of this model (4.1) is that it does not take $\alpha _t$ as an input argument. As it is “time-independent”, it only captures each point in time (from now to 10 years ago) separately. In the next section, we will propose a simple and universal model by considering time ($\alpha _t$) as a continuous argument of the outcome function *u*.

### A time-dependent mathematical model

In order to model time continuously and not just as intermittent points, we propose the following time-dependent model for the outcome function

4.5 $$\begin{aligned} u(\alpha _p, \alpha _s, \alpha _t)=\frac{c_{00} + c_{10} \alpha _p + c_{01} \alpha _s + c_{20} \alpha _p^2 +c_{11}\alpha _p\alpha _s+ c_{02} \alpha _s^2 }{a (\log {(|\alpha _t|+1)})^2+ b \log {(|\alpha _t|+1)}+1}\, , \end{aligned}$$

where $a,b,c_{00}, c_{01}, \ldots$ are constants that are to be determined as model parameters using the data. Unlike the time-independent model, this function also takes the legal parameter $\alpha _t$ as input argument and it reduces the time-independent model (4.1) at a given time. For instance, the coefficient

$$\begin{aligned} \frac{c_{10}}{a (\log {(|\alpha _t|+1)})^2+ b \log {(|\alpha _t|+1)}+1} \end{aligned}$$

denotes the importance of the factor $\alpha _p$ with respect to the passage of time. Here, we adopted the logarithmic time $\log (|\alpha _t|+1)$ for practical convenience and again kept the time-dependence up to the quadratic order. This is the most simplest choice, thus provides transparency, while satisfying the conditions we impose below. This allows us to recover the time-independent model (4.1) at any fixed time $\alpha _t$.

The model is based on the assumption that underlying facts that occur at time $\alpha _t=-\infty$ are fully covered by the right to be forgotten (Court of Justice of the European Union 2014). Consequently, ($i_1$) right to privacy would fully outweigh ($i_2$) access to information; mathematically, it means that

4.6 $$\begin{aligned} u(\alpha _p,\alpha _s,-\infty )=0 \end{aligned}$$

for all $\alpha _p$ and $\alpha _s$. Furthermore, the assumptions (see similarly, Eqs. (4.2), (4.3)) that

4.7 $$\begin{aligned} \begin{aligned} u(0,0,\alpha _t)&= 0 \text { for all } \alpha _t,\\ u(1,1,0)&= 1, \end{aligned} \end{aligned}$$

give us that $c_{00}=0$ and

4.8 $$\begin{aligned} c_{10} + c_{01} + c_{20} + c_{11} + c_{02} = 1\, . \end{aligned}$$

Again we use Mathematica to derive optimal values of the model parameters using the method of least squares. The model is rational and can be transformed to a linear optimisation problem. Thus, it is sufficient to apply the theory of linear least squares. The fitted model parameters are

4.9 $$\begin{aligned} \begin{aligned}&a^* = 0.165792, \quad b^*=-\,0.212271,\\&c^*_{01}=0.529979 , \quad c^*_{10} = -\,0.0110422, \\&c^*_{02}=-\,0.0559473, \quad c^*_{11} = 0.295508. \end{aligned} \end{aligned}$$

**Fig. 5:**
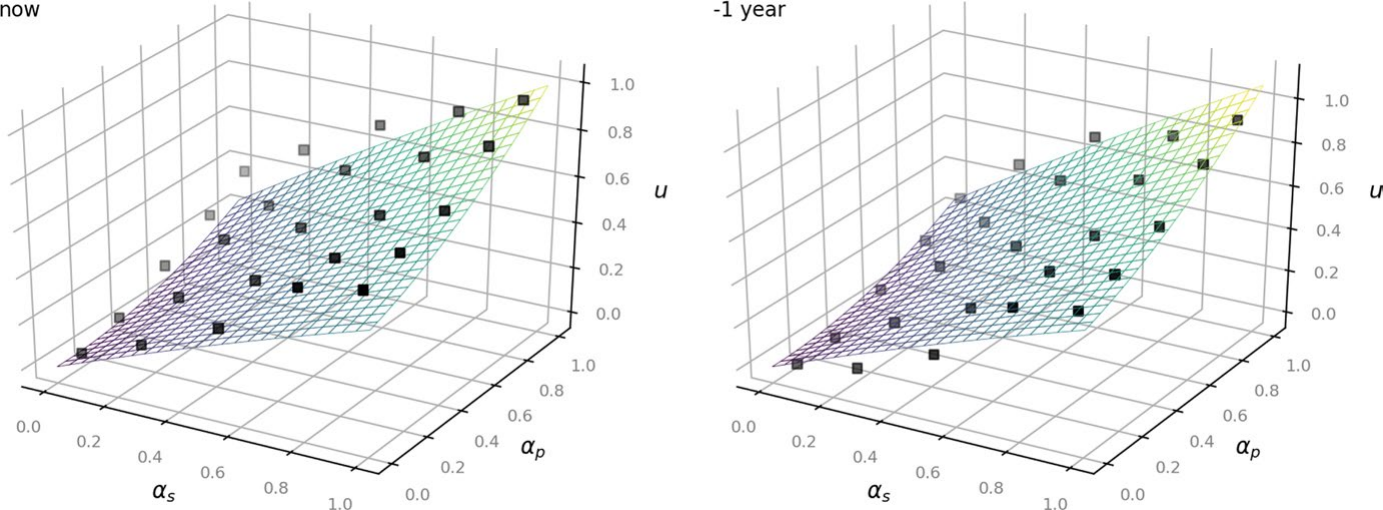
Left: now. Right:—1 year for the time-dependent model (4.5)

**Fig. 6:**
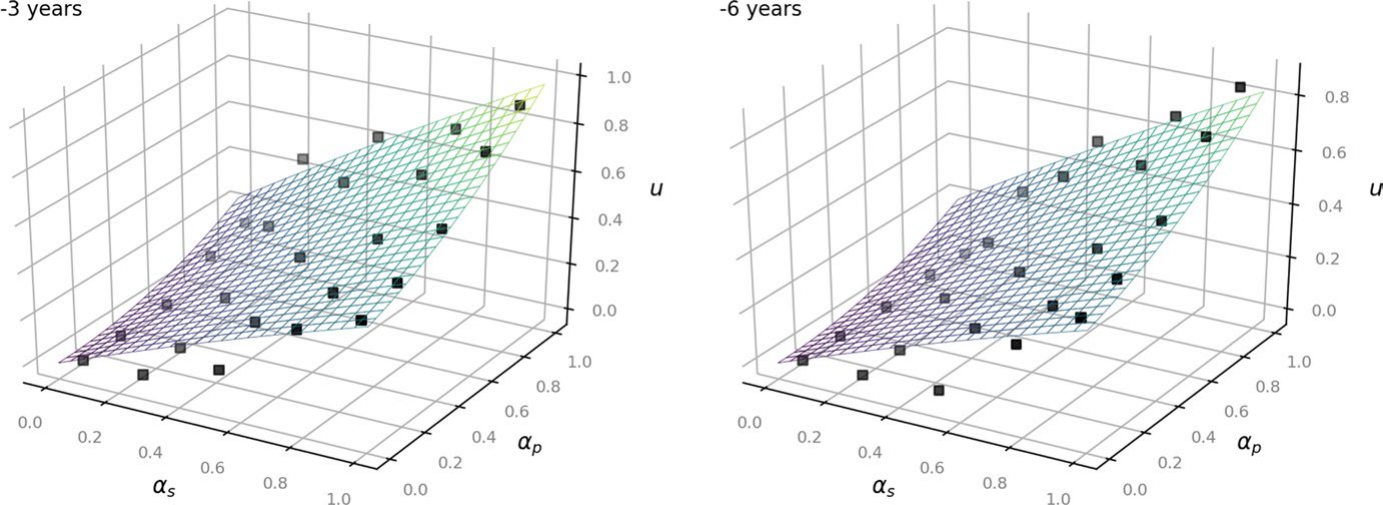
Left:—3 years. Right:—6 years for the time-dependent model (4.5)

**Fig. 7:**
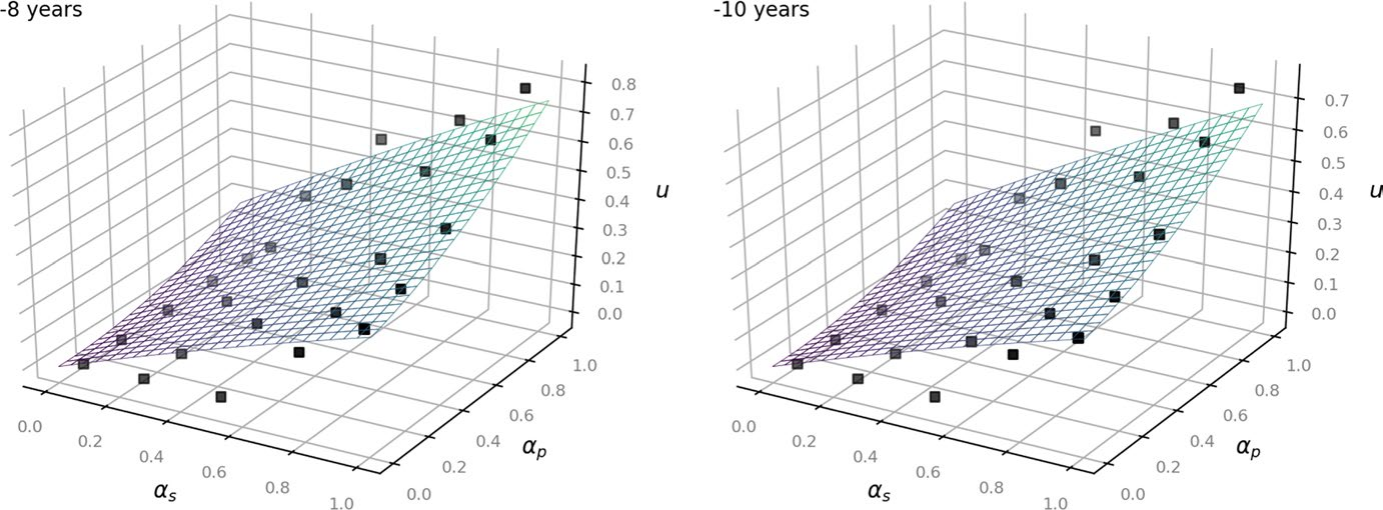
Left:—8 years. Right:—10 years for the time-dependent model (4.5)

Figs. 5, 6 and 7 illustrate the fitted time-dependent outcome function *u* as a plane in comparison to our data points for each given point in time in our data. Additionally, as the time-dependent model takes time ($\alpha _t$) as input argument, it can model any other points in time, i.e. any time in the past when the underlying facts of the respective information might have occurred.

Note that the scale for the outcome *u* decreases from Fig. 5 ($u=1.0$) to Fig 7 ($u=0.5$) corresponding to a decreasingly steep rise of the function surface. This reflects the legal consideration of the right to be forgotten, i.e. that in case of information relating to facts that occurred long time ago, the right of access to information diminishes (Art. 11 EuCH) in favor of the protection of personal data (Art. 7, Art. 8 EUCh).

## Evaluation

Finally, we turn to an evaluation of our time-dependent model. We use two quantitative evaluation methods, i.e. chi-square and cross-validation (Sects. 5.1 and 5.2), and also perform a qualitative evaluation based on a real court case (Sect. 5.3).

### Chi-square test

To evaluate the fitted function for our time-dependent model in comparison to the whole dataset, we use the chi-square test:

5.1 $$\begin{aligned} \chi ^2 := \sum ^N_{i=1} \frac{(u_\mathrm{data}-u)^2}{ u} \,, \end{aligned}$$

where *N* is the number of data in the dataset; in our case, $N=150$. This gives us the reduced chi-square

5.2 $$\begin{aligned} {\chi ^2 \over N} = 0.0343305\,. \end{aligned}$$

It implies that the fitting function can describe the original dataset with sufficient accuracy.

### Cross-validation

In order to evaluate the time-dependent model in terms of predictability, we also cross-validate our dataset. As the dataset is relatively small ($N=150$), we use leave-on-out (LOO) cross-validation. We take the absolute error between estimated outcome *u* by our fitted model and compare it to the outcome *u* from our coded data as ground truth. We measure the overall accuracy by calculating the mean absolute error (MAE) over all validation rounds:

5.3 $$\begin{aligned} MAE = 0.0728038\,. \end{aligned}$$

### Qualitative evaluation

We now turn to an evaluation of the practical application of our model to a real case. We take a judgment^1^ of the German Federal Supreme Court as a use case. The case was based on the following facts:

A was until April 2012 managing director of a regional association that organises and finances construction projects and healthcare facilities. It is with more than 500 employees and more than 35,000 members the second largest regional association of its parent in Germany. In 2011 the association had financial difficulties and a deficit of nearly one million Euro. Shortly before that, A called in sick due to health problems. Several media had reported repeatedly these facts. A wants to have the respective search engine results deleted in case his name is entered in the search engine.

The Court rejected the claim based on Art. 17 GDPR. Even after a few years, the public interest in the professional career of A would outweigh his right to data protection.

We consider the following input values as a representation of the legal criteria affecting the balancing:

**Table:** 

$\alpha _p = 0.6$	Here: regionally to statewide known person
$\alpha _s = 0.82$	Here: professional misconduct affecting a large number of people
$\alpha _t = -\,6$	Here: 6 years ago

Regarding $\alpha _t$, the underlying facts occurred in 2011 and 2012. The relevant point of time for the decision is the last judgment on the facts in 2018.^2^ Hence, $\alpha _t$ need to be $-\,6$.

If we enter these values into our time-dependent model 4.5, we get the following output for u:

5.4 $$\begin{aligned} u(0.6, 0.82, -\,6) = 0.5126022876495746\,. \end{aligned}$$

The balancing thus results in favor of access to information ($i_2$) if we define the threshold value of $u_0$ = 0.5. This corresponds to the Court’s decision to reject the claim to deletion of the respective search results.

## Discussion

Our model translated legal criteria into mathematical parameters. The data points that we assign to a specific combination of our factors can be understood as a context-dependent representation of diverging factual situations. These situations, like the circumstance of whether and to which degree a person is publicly known, influence the outcome of our balancing decision. Or in other words: the factual variance—represented by the data—is the context that affects the weight of the higher order principles that we refer to as interests. This is ultimately a question of framing and abstraction that we made a conscious decision on.^3^

Case-based reasoning approaches have used factors to model the impact of diverging arguments on a decision. Another step had been to incorporate the underlying value conflict at the root of a dialectical dispute between arguments (Bench-Capon and Prakken 2009; Bench-Capon 2003). The term “value” has been used similarly to what we refer to as “interests”. In the context of case-based reasoning, to model the impact of these “values” on the acceptance of an argument, an ordering system that ranked the preference of the respective “values” (or interests) had been suggested (Bench-Capon 2003). There is also a line of research that had formally connected factors to a comparison between conflicting values (Berman and Hafner 1993; Bench-Capon and Prakken 2009; Sartor 2018). These formalisations did not, however, use quantitative values, but required a general decision on whether a certain value should be preferred qualitatively over another one given a set of factors. The need for “quantities, not just priorities” in modeling balancing had been pointed out by Lauritsen (Lauritsen 2015). Grabmair also built on Alexy’s balancing formula (Alexy 2003) and deployed quantitative effect weights on “values” (Grabmair 2017). His approach differs from ours insofar as these weights are obtained through an iterative optimization method that grounds itself on argument schemes representing prior-defined qualitative preference relations between values and their combinatory effect on the outcome decision (Grabmair 2017). Maranhão et al. (2021) proposed an additive, i.e. linear, model of balancing.

By contrast, our models are nonlinear. They capture the preference of one interest over another depending on a continuous parameter that stands for diverging factual situations. This would bridge the gap between an abstract formalisation of a balancing decision while assuring consistency and ultimately legal certainty across cases.^4^

Our parameters are assumed to be in [0, 1], but no further assumptions are made; for instance, they could be in a non-regular subspace of [0, 1] if needed in practical applications. We also do not need to make any assumption regarding the independency of the parameters as all depends on the dataset. For instance, in the time-dependent model, the parameters $\alpha _p$ and $\alpha _s$ are time dependent, namely, they depend on time $\alpha _t$ implicitly. However, we can still fit the model in a relatively good way if a collection of the dataset $(\alpha _t,\alpha _p,\alpha _s)$ is available. From this point of view, even if $\alpha _p$ and $\alpha _s$ (or other parameters) depend on each other, the model can still work.

The prerequisite of our approach is the assumption that the legal criteria that we derive our mathematical factors from are ultimately quantifiable. This surely opens our approach up to the general critique that has been raised towards any approach that tries to formalize or quantify legal reasoning (Binns et al. 2018; Deakin and Markou 2020; Hildebrandt 2020; Martínez-Zorrilla 2018; Ronkainen 2011; Selbst et al. 2019). In light of this debate, we see our contribution in investigating and suggesting a potential design of algorithmic legal decision systems. The models certainly simplify the complexity of the underlying legal concept and would require procedural safeguards in any potential context of application (Citron 2008). But, in return, they could offer benefits for legal certainty, comparability for equal treatment and transparency in comparison to a human decision.^5^ Furthermore, the data that we chose should not be misunderstood as an ultimate decision. The outcome of the balancing ultimately depends on these data. Any change to the data would need to be justified with respect to its application and be subject to a discussion whether or how it could be incorporated in legal practice. But it would offer a method that is sensitive to the concrete case at hand and its coherence with prior cases, while generalizing well over our function. In this regard, the data may stand for the experience taken out of precedents or may represent a democratically legitimated choice of the coder.

Compared to more complex machine learning approaches our models have the advantage that they can be fitted with a relatively small dataset—150 data points in our simulations—through a simple regression algorithm, i.e. least squares. This offers higher transparency and explainability in comparison to machine learning that applies huge data sets to neural nets for model training. For simplicity we assumed simple quadratic cases, which are often sufficient in modeling many dynamic phenomena when taking computational complexity into consideration.

Finally, in the current paper, we have been focused on the simplest case of balancing two conflicting interests. To model a balancing decision between multiple interests will remain a task for future research.

## Conclusions

We proposed simple nonlinear mathematical models for the legal concept of the balancing of interests that is based on legal criteria by transforming legal criteria into arguments (or inputs) of the models. The outcome *u* was modeled as a function of the legal parameters $\alpha _p$ (status of a person), $\alpha _s$ (sphere of information) and $\alpha _t$ (time). The models thus capture the preference of one interest over another depending on a continuous parameter that stands for diverging factual situations. The model parameters were optimised via the method of least squares, by making use of the dataset. The evaluation via the chi-square test shows that our models can sufficiently describe the original data.

While the proposed models certainly do not equal an actual legal decision in terms of considering all relevant legal criteria, the particularities and complexity of the case at hand, and in terms of legal protection through legal procedure, we believe it makes valuable contributions at a conceptional and methodological level: in investigating to which extent and how legal assessment could mathematically be modeled, in mirroring the impact of legal criteria for a balancing decision, and in the role of the data needed to fit the function. The models offer an abstract formalisation of a balancing decision while assuring consistency and ultimately legal certainty across concrete cases. By comparison to other approaches based on machine learning, especially neural networks, this approach requires significantly less data (here: 150 number of data points). This might come at the price of higher abstraction and simplification, but also provides for higher transparency and explainability.

### Supplementary Information

Below is the link to the electronic supplementary material.

Supplementary file1 (XLSX 11 kb)

## Footnotes

1 BGH, 27.07.2020, VI ZR 405/18.

2 OLG Frankfurt, 06.09.2018, I-16 U 193/17, 16 U 193/17.

3 Dancy (Dancy 2006) had argued that “values” may be context-dependent and thus be influenced by other “values”. In our model, however, the “values” that Dancy refers to are the interests that we balance against each other. Our parameters, on the other side, may be dependent or independent conditional to the a dataset.

4 Sartor had argued for “consistency in balancing” for value-based decisions with regard to precedents (Sartor 2018).

5 See for human heuristics and biases in legal decision-making: (Glöckner and Engel 2013; Guthrie et al. 2001; Simon 2004; Weinstein 2002).
